# Growth Inhibitory Effects of Ester Derivatives of Menahydroquinone-4, the Reduced Form of Vitamin K_2(20)_, on All-Trans Retinoic Acid-Resistant HL60 Cell Line

**DOI:** 10.3390/pharmaceutics13050758

**Published:** 2021-05-20

**Authors:** Hirofumi Yamakawa, Shuichi Setoguchi, Shotaro Goto, Daisuke Watase, Kazuki Terada, Nami Nagata-Akaho, Erina Toki, Mitsuhisa Koga, Kazuhisa Matsunaga, Yoshiharu Karube, Jiro Takata

**Affiliations:** Faculty of Pharmaceutical Sciences, Fukuoka University, Nanakuma, Jonan-ku, Fukuoka 814-0180, Japan; hyamakawa@adm.fukuoka-u.ac.jp (H.Y.); ssetoguchi@fukuoka-u.ac.jp (S.S.); sgoto@fukuoka-u.ac.jp (S.G.); watase@fukuoka-u.ac.jp (D.W.); kterada@fukuoka-u.ac.jp (K.T.); nanohana_73@jewel.ocn.ne.jp (N.N.-A.); pd191005@cis.fukuoka-u.ac.jp (E.T.); kogami@fukuoka-u.ac.jp (M.K.); k-matsu@fukuoka-u.ac.jp (K.M.); karube@fukuoka-u.ac.jp (Y.K.)

**Keywords:** drug delivery, HL60, leukemia, prodrug, resistance, retinoic acid, vitamin K

## Abstract

The first-choice drug for acute promyelocytic leukemia (APL), all-trans retinoic acid (ATRA), frequently causes drug-resistance and some adverse effects. Thus, an effective and safe agent for ATRA-resistant APL is needed. Menaquinone-4 (MK-4, vitamin K_2(20)_), used for osteoporosis treatment, does not have serious adverse effects. It has been reported that MK-4 has growth-inhibitory effects on HL60 cells by inducing apoptosis via the activation of Bcl-2 antagonist killer 1 (BAK). However, the effect of MK-4 on ATRA-resistant APL has not been reported. Here, we show that ester derivatives of menahydroquinone-4 (MKH; a reduced form of MK-4), MKH 1,4-bis-*N*,*N*-dimethylglycinate (MKH-DMG) and MKH 1,4-bis-hemi-succinate (MKH-SUC), exerted strong growth-inhibitory effects even on ATRA-resistant HL60 (HL-60R) cells compared with ATRA and MK-4. MKH delivery after MKH-SUC treatment was higher than that after MK-4 treatment, and the results indicated apoptosis induced by BAK activation. In contrast, for MKH-DMG, reconversion to MKH was slow and apoptosis was not observed. We suggest that the ester forms, including monoesters of MKH-DMG, exhibit another mechanism independent of apoptosis. In conclusion, the MKH derivatives (MKH-SUC and MKH-DMG) inhibited not only HL60 cells but also HL-60R cells, indicating a potential to overcome ATRA resistance.

## 1. Introduction

Acute promyelocytic leukemia (APL) is a unique subtype of acute myeloid leukemia (AML). All-trans retinoic acid (ATRA), also known as tretinoin, a molecularly targeted drug, has been used for the initial (induction) treatment of APL in combination with anthracyclines. The cure rate associated with this general treatment approach is 80–90%. However, it has been reported that approximately 15–20% of patients relapse and most of them acquire ATRA resistance [1,2,3,4,5]. Thus, there is a demand for effective and safe agents for ATRA-resistant APL. HL60 was initially classified as an APL cell line, and ATRA was found to induce its differentiation; this discovery led to numerous studies [6,7]. HL60 was later reclassified as a non-APL cell line [8]. As mentioned below, because the aim of this study was to discover drug candidates that potentially overcome ATRA resistance, we used HL60 and HL-60R (ATRA-resistant HL60) as ATRA-responsive cell lines.

It has been reported that menaquinone-4 (MK-4, vitamin K_2(20)_) induces apoptosis in leukemia cell lines and primary cultured leukemia cells. MK-4 shows greater inhibitory effects on myeloid cells such as myeloblasts, which are abnormal promyelocytes, than on lymphoid cells. Therefore, MK-4 may exert selective effects against APL cells without any adverse effects on normal bone marrow cells in patients with APL [9,10,11,12,13,14,15,16]. MK-4 has been used to treat osteoporosis, especially in postmenopausal women, and its long-term safety has been established. Thus, MK-4 has the potential to be an alternative chemotherapeutic agent for ATRA. However, the effect of MK-4 against ATRA-resistant APL has not been reported.

**Figure 1:**
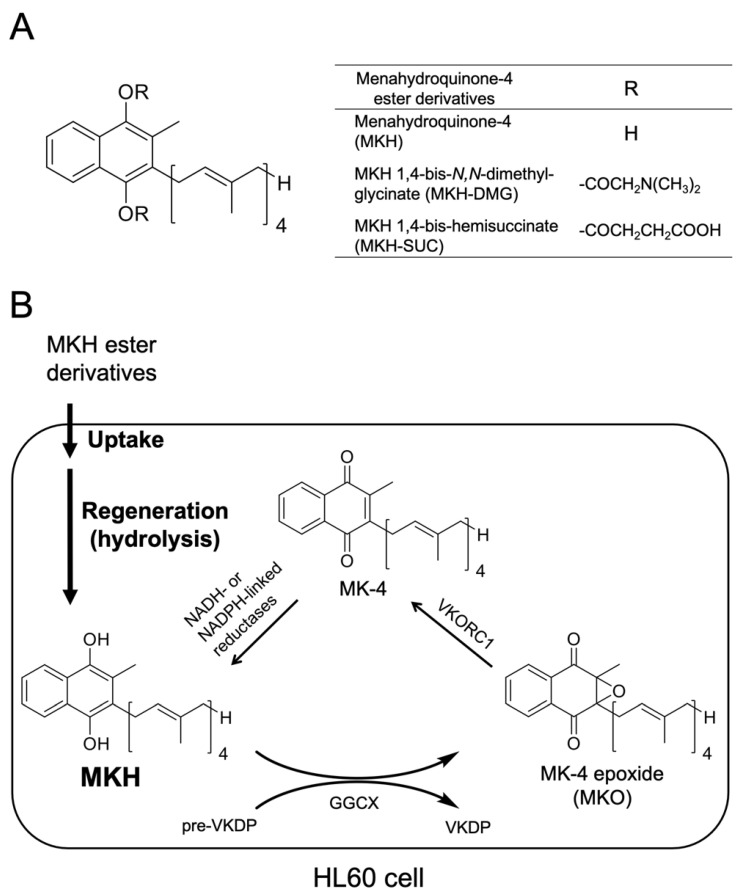
Chemical structure of MKH derivatives (**A**). The activation step for MKH derivatives, and the vitamin K cycle in HL60 cells (**B**). MKH, menahydroquinone-4; MK-4, menaquinone-4; MKO, menaquinone-4 epoxide; VKR, vitamin K reductase; VKOR, vitamin K epoxide reductase; VKDP, vitamin K-dependent proteins; GGCX, γ-glutamyl carboxylase.

MK-4 is reduced to menahydroquinone-4 (MKH) via the vitamin K cycle in cells. MKH is a cofactor of γ-glutamyl carboxylase (GGCX), which converts glutamic acid residues into γ-carboxyglutamic acid residues of vitamin K-dependent proteins. In other words, the post-translational modification of vitamin K-dependent proteins depends on the supplied MKH. When MKH acts as a cofactor, it is stoichiometrically converted to MK-4 epoxide (MKO), which is further reduced to MK-4 for reuse (Figure 1B).

It has been reported that MK-4 induces apoptosis via the activation of BAK, a member of the Bcl-2 family of apoptosis-promoting proteins, indicating an interaction between MKO and BAK [12]. Previously, we reported that the MKH ester derivatives 1,4-bis-*N*,*N*-dimethylglycinate (MKH-DMG) and 1,4-bis-hemi-succinate (MKH-SUC) (Figure 1A) act as prodrugs for hepatocellular carcinoma (HCC) cells, and exhibit strong antitumor effects compared with MK-4 [17,18]. As MKH is the active form of MK-4, in terms of the physiological effects, such as blood coagulation and bone formation, MKH may contribute to the inhibitory effect of MK-4. In this study, we investigated the efficacy of MKH ester derivatives against ATRA-responsive HL60 and ATRA-resistant HL60 (HL-60R) cells, the delivery of MKH by these derivatives, and the underlying mechanisms.

## 2. Materials and Methods

### 2.1. Chemicals

MK-4 and MKO were kindly provided by Eisai (Tokyo, Japan). MKH-DMG and MKH-SUC were synthesized in our laboratory, and the nuclear magnetic resonance (NMR) spectra and mass spectra for MKH-DMG and MKH-SUC have been reported previously [18,19,20]. ATRA and other chemicals were purchased from FUJIFILM Wako Pure Chemical (Osaka, Japan) unless otherwise specified.

### 2.2. Cell Lines

Human promyelocytic cell lines HL60 (RCB0041) and ATRA-resistant HL-60R (RCB1550, derived from RCB0041) were provided by RIKEN BRC (Ibaraki, Japan) [21]. The cell lines were maintained in RPMI 1640 (FUJIFILM Wako Pure Chemical) supplemented with 10% fetal bovine serum (Nichirei Biosciences, Tokyo, Japan) and 1% penicillin/streptomycin (Life Technologies, Carlsbad, CA, USA) at 37 °C under an atmosphere of 5% CO_2_.

### 2.3. Cell Viability Assay

HL60 and HL-60R cells were seeded at 3.0 × 10^3^ cells/well in 96-well plates and incubated for 24 h. ATRA, MK-4, MKH-DMG, and MKH-SUC were initially dissolved in ethanol. Stock solutions (15 mM) of each drug were diluted to the intended final concentrations with medium. Cells were exposed to a medium containing ATRA, MK-4, MKH-DMG, or MKH-SUC for 72 h, and cell viability was measured using the CellTiter-Glo 2.0 Luminescent Cell Viability Assay kit (Promega, Madison, WI, USA), according to the manufacturer’s instructions. The CellTiter-Glo assay detects intracellular ATP, thereby enabling identification of living cells. The doubling times of the HL60 and HL-60R cells using CellTiter-Glo were 24.9 ± 3.68 and 26.2 ± 2.69 h, respectively. These values were almost consistent with the number of cells counted by staining them with trypan blue after each subculturing. The IC_50_ values were determined using a log (drug) vs. normalized response-variable slope analysis with GraphPad Prism, version 6.0 (GraphPad Software, San Diego, CA, USA). Resistance index was calculated using the following equation:

Resistance index = IC_50_ to HL-60R/IC_50_ to HL60 (1)

### 2.4. Determination of Intracellular MK-4 and MKO Levels after Drug Treatment

HL60 and HL-60R cells were seeded at 3.0 × 10^5^ cells/well in six-well plates and incubated for 24 h. The cells were cultured in a medium containing MK-4, MKH-DMG, or MKH-SUC for the indicated time. After drug exposure, the medium was removed, and the cells were washed twice with PBS. Intracellular drug extraction was performed as previously described [22], and cell protein concentration was determined using a BCA protein assay kit (Thermo Fisher Scientific, Waltham, MA, USA). Curve fitting was performed using a Michaelis–Menten slope analysis with GraphPad Prism, version 6.0 (GraphPad Software).

### 2.5. LC-MS/MS

The LC-MS/MS system and conditions were set as previously described [22]. Briefly, LC-MS/MS was performed using an LCMS-8050 and Shimadzu UFLC System (Shimadzu, Kyoto, Japan) with a CAPCELL PAK C18 UG120 column (3 μm, 2.0 mm × 100 mm; Shiseido Co., Ltd., Tokyo, Japan) and mobile phase of 10 mM ammonium acetate and 0.1% acetic acid in methanol and water (97:3) at a flow rate of 0.4 mL/min. Identification and quantitation were performed in the MS/MS-multiple reaction monitoring (MRM) mode with positive electrospray ionization. During MRM, *m*/*z* was 445→187 for the [M + H]^+^ MK-4 adduct and 461→81 for the [M + H]^+^ MKO adduct. The retention times were 3.3 min for MK-4 and 2.5 min for MKO.

### 2.6. Cell Cycle Analysis

HL60 and HL-60R cells were seeded at 3.0 × 10^5^ cells/dish in 100 mm dishes and incubated for 24 h. The cells were then exposed to medium containing ATRA, MK-4, MKH-DMG, or MKH-SUC for 72 h. The medium was removed, and the cells were washed with PBS and fixed in 70% ethanol at −20 °C overnight. The supernatant was removed after centrifugation, and the cell pellet was resuspended in 500 µL of RNase (0.25 mg/mL; Sigma Aldrich, St. Louis, MO, USA) and incubated for 30 min at 37 °C. The suspension was treated with 10 µL of propidium iodide (PI, 1 mg/mL) on ice for 20 min. Cell populations and cell cycle phases were analyzed using a JSAN flow cytometer (Bay Bioscience, Kobe, Japan) and FlowJo software (Becton Dickinson, Franklin Lakes, NJ, USA).

### 2.7. Apoptosis Detection

HL60 and HL-60R cells were seeded at 3.0 × 10^5^ cells/dish in 100 mm dishes and incubated for 24 h. The cells were exposed to a medium containing MK-4, MKH-DMG, or MKH-SUC for 72 h. Apoptotic cells were detected using an Annexin V-FITC Apoptosis Detection kit (Medical & Biological Laboratories, Aichi, Japan) according to the manufacturer’s instructions. The percentage of apoptotic cells to total cell population in each sample was determined using a JSAN flow cytometer (Bay Bioscience). Cells simultaneously stained negative for Annexin V-FITC and PI were considered live cells. Cells stained positive for Annexin V-FITC were considered apoptotic cells, and PI-negative and -positive cells were considered to be early- and late-stage apoptotic, respectively.

### 2.8. Mitochondrial Membrane Potential

HL60 and HL-60R cells were seeded at 1.0 × 10^5^ cells/well in 12-well plates and incubated for 24 h. The cells were exposed to a medium containing MK-4, MKH-DMG, or MKH-SUC for 48 h. Mitochondrial membrane potential was detected using the lipophilic cationic probe JC-1 (DOJINDO, Tokyo, Japan). The cells were washed with culture medium, and then incubated in culture medium containing 2 µM JC-1 for 30 min at room temperature in the dark. Next, the cells were washed twice with the imaging buffer provided in the JC-1 package, and the mitochondrial membrane potential was determined using a JSAN flow cytometer (Bay Bioscience).

### 2.9. Determination of Cytochrome C in Mitochondrial and Cytosolic Fractions

HL60 and HL-60R cells were seeded at 1.0 × 10^5^ cells/well in six-well plates and incubated for 24 h. Next, the cells were treated with or without 10 μM MK-4, MKH-DMG, or MKH-SUC for 48 h. The cells were washed three times with ice-cold PBS, resuspended in isotonic buffer A (10 mM HEPES, 0.3 M mannitol, and 0.1% BSA) with 0.1 mM digitonin, left on ice for 5 min, and centrifuged at 8500× *g* for 5 min at 4 °C. The supernatant was used as the cytosolic fraction. The pellet was resuspended in sonication buffer (50 mM Tris-HCl, pH 7.4, 150 mM NaCl, 2 mM EDTA, 1 mM PMSF, and 0.5% Tween 20), sonicated twice on ice, and centrifuged at 10,000× *g* for 30 min at 4 °C. The supernatant was used as the mitochondrial fraction. The amount of cytochrome c in the mitochondrial and cytosolic fractions was measured using the cytochrome c ELISA kit (R&D Systems, Minneapolis, MN, USA) according to the manufacturer’s instructions. The cell protein concentration was determined using a BCA protein assay kit.

### 2.10. Western Blotting

HL60 and HL-60R cells were seeded at a density of 1.5 × 10^5^ cells/well in six-well plates for 36 h. Next, the cells were treated with or without 10 μM MK-4, MKH-DMG, or MKH-SUC for 24, 48, or 72 h. The cells were washed with PBS after removing the drug-containing medium and lysed with RIPA buffer (0.5% NP-40, 0.25% sodium deoxycholate, 0.05% SDS, 150 mM NaCl, and 50 mM HEPES, pH 7.4) containing a protease inhibitor cocktail (Nacalai Tesque, Kyoto, Japan). Each cell lysate was combined with sample loading buffer (Nacalai Tesque), separated on 15% SDS-PAGE gels (SuperSep Ace, FUJIFILM Wako), and transferred onto PVDF membranes (BIO-RAD, Hercules, CA, USA). After blocking, the membranes were incubated with the following primary antibodies: anti-pro/p17-caspase-3, anti-cleaved PARP1 (1:2000) (ab136812; Abcam, Cambridge, UK), rabbit anti-Bak (1:2000) (#12105; Cell Signaling Technology, Danvers, MA, USA), and mouse anti-GAPDH antibody (1:2000) (G8795; Sigma-Aldrich, St. Louis, MO, USA). After washing, the membranes were treated with appropriate secondary antibodies and visualized using Immunostar LD or Immunostar Zeta (FUJIFILM Wako).

### 2.11. Statistical Analysis

The statistical significance of the results in Section 3.1 was determined using the extra sum-of-squares F test, and that of the results in the other sections was determined using Dunnett’s test. All analyses were carried out using GraphPad Prism 6 (GraphPad Software, San Diego, CA, USA); results with *p* < 0.05 were considered significant.

## 3. Results

### 3.1. Growth-Inhibitory Effects of MKH Derivatives on HL60 and HL-60R Cells

**Figure 2:**
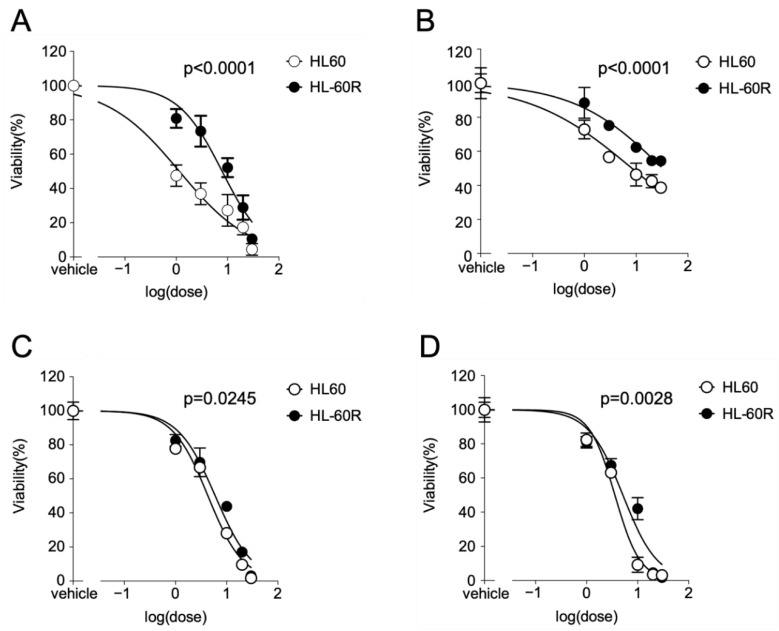
Inhibitory effects of ATRA, MK-4, and MKH derivatives on the viability of HL60 and HL-60R cells. Cells were treated with 1–30 μM ATRA (**A**), MK-4 (**B**), MKH-DMG (**C**), or MKH-SUC (**D**) for 72 h. Plotted values are mean ± SD (n = 3). *p* values were analyzed using the extra sum-of-squares F test.

To assess the growth inhibitory effects of MKH derivatives (MKH-DMG and MKH-SUC) on HL60 and HL-60R cell lines, a dose–cell viability plot at 72 h after treatment with the test drugs was generated (Figure 2). The curves of viable HL60 and HL60R cells after drug treatment were compared using the extra sum-of-squares F test. All test drugs reduced the viability of both cell lines in a dose-dependent manner. The IC_50_ values and resistance indexes are summarized in Table 1. As the integrities of the curves from the MK-4 treatments are insufficient to determine IC_50_ values, the obtained *p* values and IC_50_ values for MK-4 were only used for reference. For ATRA treatment, the viability curve of the HL-60R cells shifted towards the higher doses represented on the HL60 cell curve (Figure 2A). Similarly, for the MK-4 treatment, the curve of the HL-60R cells shifted towards the higher doses for HL60 cells (Figure 2B). In contrast, the MKH-DMG and MKH-SUC treatments strongly inhibited the growth of both cell lines in a dose-dependent manner compared with MK-4 (Figure 2C,D). The resistance index values of MKH-DMG and MKH-SUC were considerably lower than those of ATRA and MK-4 and were close to 1 (Table 1). Similarly, to assess the growth-inhibitory effect of MKH-DMG and MKH-SUC on the APL cell line NB-4, a dose–cell viability plot at 72 h after treatment with the test drugs was generated (Supplementary Figure S1). MKH-DMG and MKH-SUC decreased the viability of cells in a dose-dependent manner. The IC_50_ value for MKH-DMG was 16.9 µM (95% CI: 14.4–20.0) and that for MKH-SUC was 10.2 µM (95% CI: 9.50–10.9).

### 3.2. Intracellular Drug Levels in HL60 and HL-60R Cells Treated with MK-4 and MKH Derivatives

**Figure 3:**
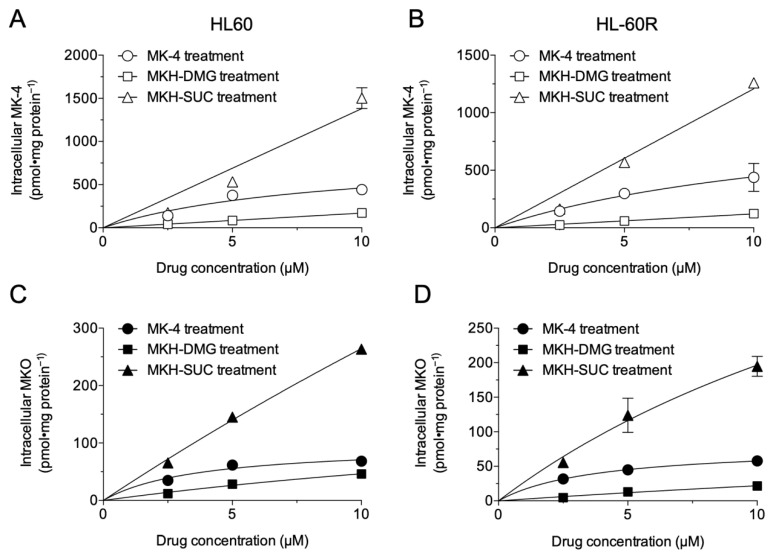
Intracellular level of MK-4 and MKO in HL60 and HL-60R cells treated with MK-4 or MKH derivatives. Intracellular MK-4 in HL60 (**A**) and HL-60R (**B**) cells treated with 2.5–10 μM MK-4, MKH-DMG, or MKH-SUC for 24 h, and intracellular MKO in HL60 (**C**) and HL-60R (**D**) cells treated with 2.5–10 μM MK-4, MKH-DMG, or MKH-SUC for 24 h. Plotted values are mean ± SD (n = 3).

To determine the intracellular drug levels corresponding to the growth inhibitory effects, cells treated with the test drugs were evaluated by LC-MS/MS (Figure 3). The intracellular levels of MK-4 and MKO in HL60 and HL-60R cells after 24 h of treatment with 2.5–10 μM MK-4, MKH-DMG, or MKH-SUC are shown in Figure 3. Each dose–intracellular drug concentration plot is presented with curve fitting using the Michaelis–Menten model. When quantifying intracellular MKH levels, intracellular MKH was detected as MK-4, the oxidized reactant of MKH (reduced form), and intracellular MKO reflected the amount of MKH delivered, as MKH is stoichiometrically converted to MKO when it functions as a cofactor for GGCX (Figure 1B). Thus, both intracellular MK-4 and MKO levels after treatment with MK-4 or MKH derivatives were regarded as indicators of MKH delivery. All test drugs increased the intracellular MK-4 and MKO levels in a dose-dependent manner in both HL60 and HL-60R cell lines (Figure 3A–D). The intracellular MK-4 (Figure 3A,B) and MKO (Figure 3C,D) levels in cells treated with MK-4 reached a plateau at 5 μM in both cell lines. In contrast, the saturation of the intracellular MK-4 (Figure 3A,B) and MKO (Figure 3C,D) levels in cells treated with MKH-DMG or MKH-SUC was not observed, and these levels increased in a dose-dependent manner in this dose range (2.5–10 μM). The intracellular MK-4 and MKO levels after MKH-SUC treatment were higher than those after MK-4 treatment. In contrast, the intracellular MK-4 and MKO levels after MKH-DMG treatment were lower than those after MK-4 treatment.

**Figure 4:**
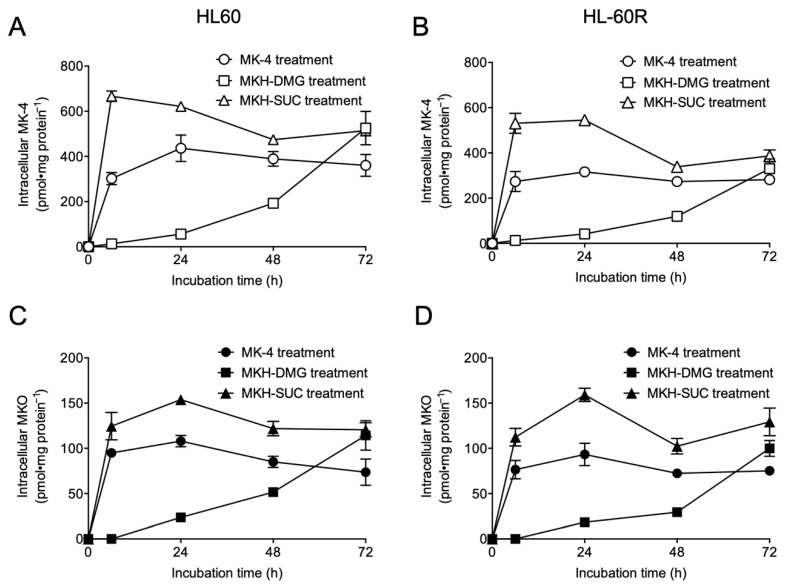
Intracellular concentration of MK-4 and MKO in HL60 and HL-60R cells treated with MK-4 or MKH derivatives. Intracellular MK-4 levels in HL60 (**A**) and HL-60R (**B**) cells treated with 5 μM MK-4, MKH-DMG, or MKH-SUC up to 72 h, and intracellular MKO levels in HL60 (**C**) and HL-60R (**D**) cells treated with 5 μM MK-4, MKH-DMG, or MKH-SUC up to 72 h. Plotted values are mean ± SD (n = 3).

The time course of intracellular MK-4 and MKO levels up to 72 h of treatment with 5 μM of each drug is shown in Figure 4. The drug concentration was set at 5 µM, which is an approximation of the IC_50_ values of the MKH derivatives given in Table 1. The intracellular MK-4 (Figure 4A,B) and MKO (Figure 4C,D) levels after MK-4 or MKH-SUC treatment rapidly increased and reached a plateau at 24 h. The intracellular MK-4 and MKO levels after MKH-SUC treatment were higher than those after MK-4 treatment at earlier time points, and the AUC_0–72h_ for MK-4 or MKO after MKH-SUC treatment was approximately 1.5-fold higher than that for MK-4 in both cell lines (Table 2). In contrast, the intracellular MK-4 and MKO levels after MKH-DMG treatment steadily increased in a time-dependent manner and surpassed those of MK-4 at 72 h. The AUC_0–72h_ for MK-4 or MKO after MKH-DMG treatment was approximately half that after MK-4 treatment in both cell lines (Table 2). However, the intracellular MKH-DMG level after MKH-DMG treatment increased rapidly in both cell lines, reaching a peak at 24 h. Furthermore, the sequential hydrolysis reaction of MKH-DMG was observed, indicating the elimination of bis-ester and the generation of the corresponding monoesters in a time-dependent manner (Supplementary Figure S2).

### 3.3. Effects of MKH Derivatives on the Cell Cycle of HL60 and HL-60R Cells

**Figure 5:**
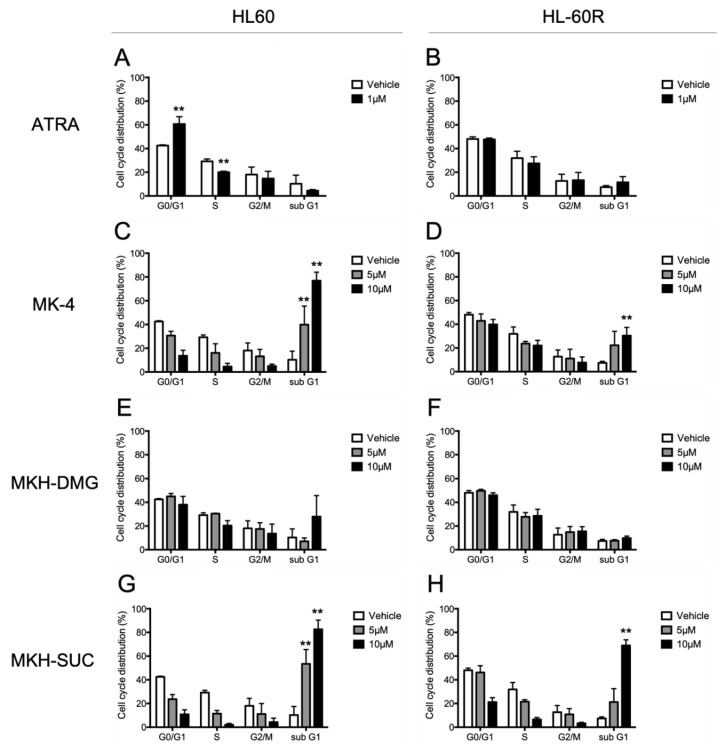
Effects of ATRA, MK-4, and MKH derivatives on the cell cycle distribution of HL60 and HL-60R cells. HL60 (**A**,**C**,**E**,**G**) and HL-60R (**B**,**D**,**F**,**H**) cells were treated with 1 μM ATRA (**A**,**B**), 5 or 10 μM MK-4 (**C**,**D**), 5 or 10 μM MKH-DMG (**E**,**F**), or 5 or 10 μM MKH-SUC (**G**,**H**) for 72 h. Cell cycle distribution was analyzed by flow cytometry after PI staining. Data are shown as mean ± SD from triplicate experiments. ** *p* < 0.01 by Dunnett’s test.

To determine the effects of the test drugs on the cell cycle, we performed flow cytometry with PI staining (Figure 5). The distributions at each cell cycle phase in HL60 and HL-60R cells treated with 1 μM ATRA, the pharmacological dose, for 72 h areis shown in Figure 5A,B, respectively. The percentage of ATRA-treated HL60 cells in the G_0_/G_1_ phase significantly increased, whereas that in the S phase decreased, indicating G_1_ arrest. In contrast, G_1_ arrest was not observed in HL-60R cells treated with ATRA (Figure 5B). The distributions at each cell cycle phase in HL60 and HL-60R cells treated with 5 and 10 μM MK-4 or MKH derivatives for 72 h are shown in Figure 5C–H. In the MK-4 treatment, G_1_ arrest was not observed in either cell line. The percentage of HL60 cells in the sub-G_1_ phase, which indicates DNA fragmentation by apoptosis, significantly increased (Figure 5C). On the other hand, the percentage of HL-60R cells in the sub-G_1_ phase slightly increased compared with that of the HL60 cells (Figure 5D). In the MKH-DMG treatment, there was no change in cell distribution at either the G_0_/G_1_–S or the sub-G_1_ phase in either cell line (Figure 5E,F). Under the MKH-SUC treatment, G_1_ arrest was not observed, but the sub-G_1_ cell distribution in both cell lines significantly increased (Figure 5G,H), similarly to that under the MK-4 treatment. The percentage of HL-60R cells in the sub-G_1_ phase was considerably higher under the 10 μM MKH-SUC treatment than under the 10 μM MK-4 treatment (Figure 5D,H).

### 3.4. Effects of MKH Derivatives on the Cell Apoptosis of HL60 and HL-60R Cells

**Figure 6:**
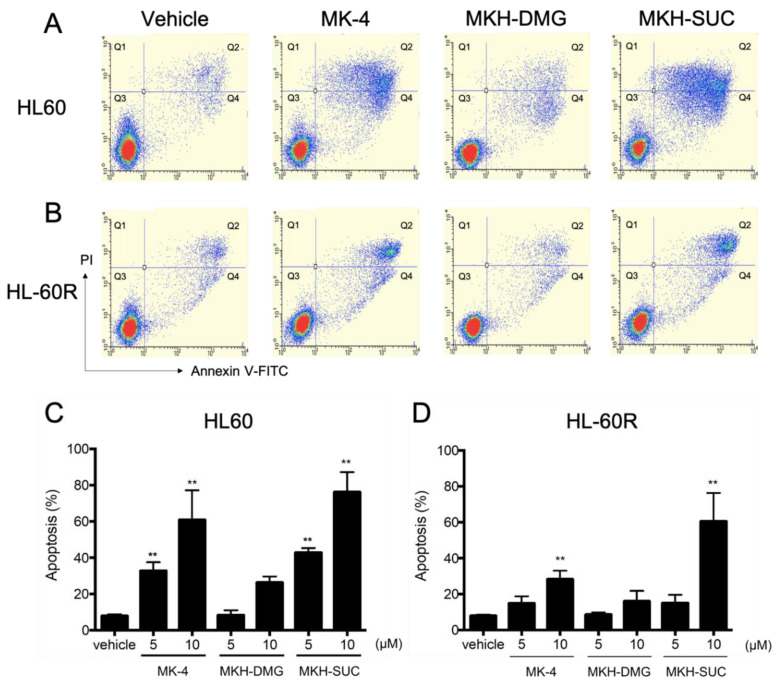
Effects of MK-4 and MKH derivatives on the apoptosis in HL60 and HL-60R cells. Typical flow cytograms of HL60 (**A**) and HL-60R (**B**) cells treated with the test drugs at 5 µM for 72 h are shown, with percentages of apoptotic HL60 (**C**) and HL-60R (**D**) cells treated with 5 or 10 μM MK-4, MKH-DMG, or MKH-SUC for 72 h. Apoptotic cells were determined using flow cytometry after Annexin V/PI staining. Data are shown as mean ± SD from triplicate experiments. ** *p* < 0.01 vs. vehicle by Dunnett’s test.

To assess the cell apoptosis involved in the growth inhibitory effect of MKH derivatives on HL60 and HL-60R cells, apoptotic cells were detected using flow cytometry with Annexin V/PI staining (Figure 6). The typical flow cytograms for HL60 and HL-60R cells are shown in Figure 6A,B, respectively. Following treatment with MK-4 or MKH-SUC, the number of apoptotic HL60 (Figure 6C) and HL-60R (Figure 6D) cells increased in a dose-dependent manner. Under the MKH-DMG treatment, apoptotic cells tended to increase at 10 μM, but not at 5 μM (Figure 6C,D). Ten micromolar of MKH-SUC induced strong apoptosis in both cell lines (Figure 6C,D).

### 3.5. Effect of MKH Derivatives on Mitochondrial Apoptotic Pathway

To confirm whether the apoptosis of cells induced by the MKH derivatives involves the mitochondrial pathway, mitochondrial membrane potential, cytochrome c release, and expression of apoptosis-related proteins (pro-/cleaved caspase-3, cleaved PARP, and BAK) were analyzed using flow cytometry with JC-1 staining, ELISA, and Western blotting, respectively.

**Figure 7:**
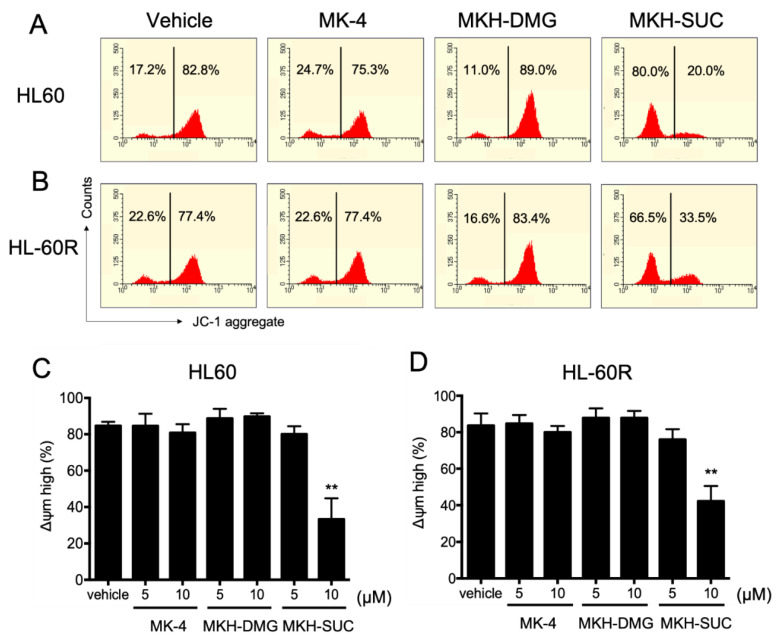
Effects of MK-4 and MKH derivatives on the mitochondrial membrane potentials of HL60 and HL-60R cells. Typical flow cytograms of HL60 (**A**) and HL-60R (**B**) cells treated with the test drugs at 10 µM for 48 h. Percentages of ΔΨm-high cells among HL60 (**C**) and HL-60R (**D**) cells treated with 5 or 10 μM MK-4, MKH-DMG, or MKH-SUC for 48 h. The mitochondrial membrane potentials were determined using flow cytometry after JC-1 staining. Data are shown as mean ± SD from triplicate experiments. ** *p* < 0.01 vs. vehicle by Dunnett’s test.

**Figure 8:**
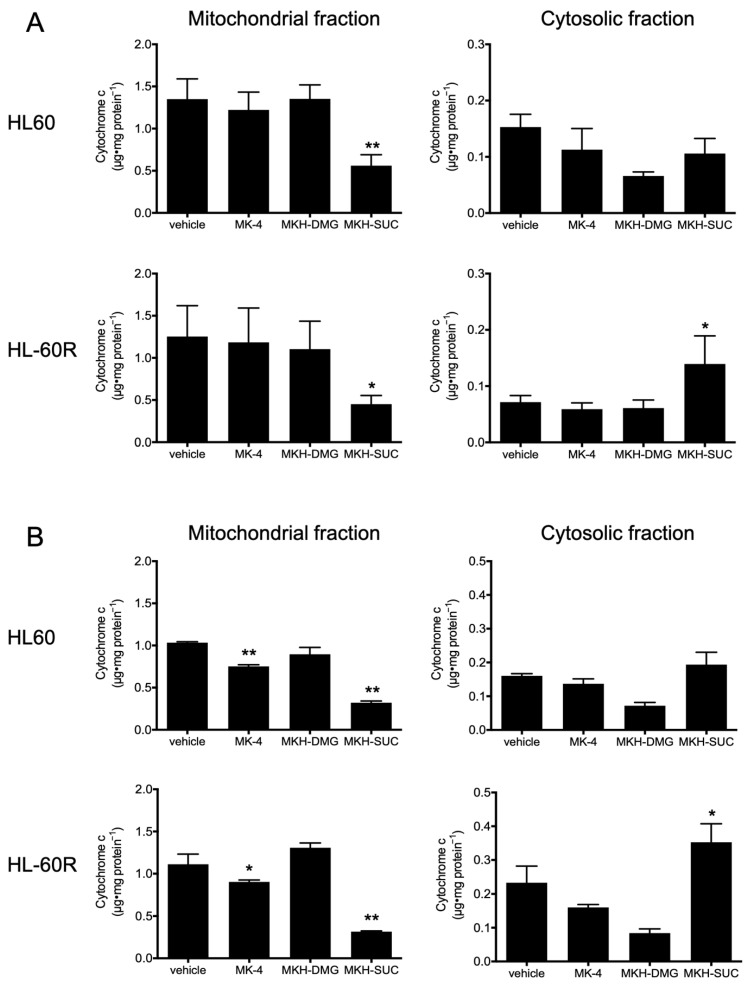
Release of cytochrome c in HL60 and HL-60R cells treated with MK-4 and MKH derivatives. HL60 and HL-60R cells treated with the test drugs at 10 µM for 48 h (**A**) or 72 h (**B**). The cytochrome c level in the mitochondrial or cytosolic fractions was determined using ELISA. Data are shown as mean ± SD from at least 3 replicates. * *p* < 0.05, ** *p* < 0.01 vs. vehicle by Dunnett’s test.

**Figure 9:**
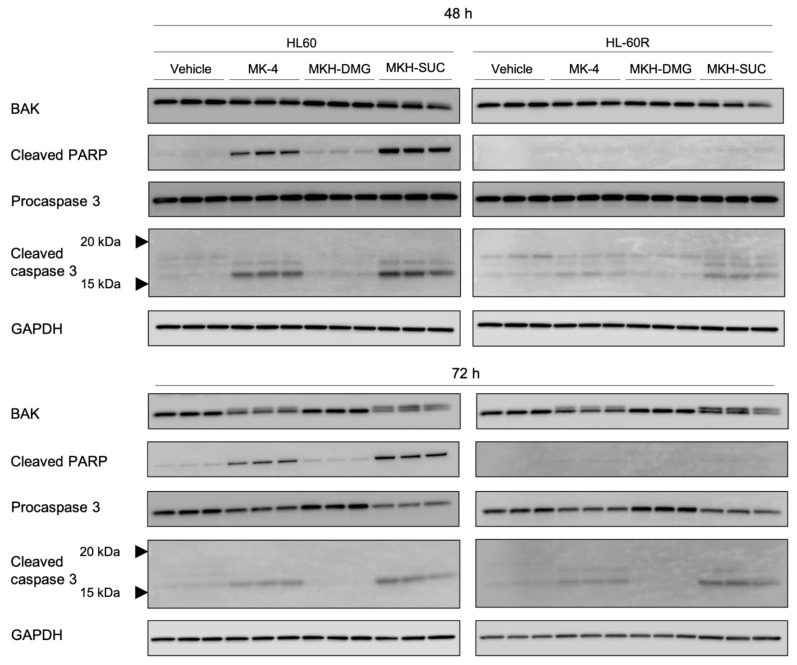
Effects of MK-4 and MKH derivatives on the expression of apoptosis-related proteins in HL60 and HL-60R cells. HL60 and HL-60R cells were treated with 10 µM MK-4, MKH-DMG, or MKH-SUC for 48 and 72 h. Th expression of BAK, cleaved PARP, and pro/cleaved caspase-3 was analyzed by Western blotting.

The mitochondrial membrane potential of both cell lines treated with 10 μM MK-4 and 5 µM MKH-SUC for 48 h tended to decrease slightly compared with that under treatment with the vehicle, and that of cells treated with 10 μM MKH-SUC strongly decreased (Figure 7C,D). In contrast, the MKH-DMG treatment did not affect the potentials in either cell line (Figure 7C,D). The level of mitochondrial cytochrome c in both cell lines treated with 10 μM MK-4 decreased at 48 h and those in cells treated with MKH-SUC decreased at both 48 and 72 h (Figure 8). In the cytosolic fraction, the cytochrome c level after MK-4 treatment did not increase, whereas that in HL-60R cells significantly increased after MKH-SUC treatment for 72 h (Figure 8). In contrast, MKH-DMG did not promote the release of cytochrome c from the mitochondria to the cytosol (Figure 8). The Western blotting analysis showed that the bands of modified BAK protein in both cell lines treated with 10 μM MK-4 and MKH-SUC for 48 h migrated at a slightly higher molecular weight than the bands of free BAK. Moreover, this doublet was clearly observed following MK-4 and MKH-SUC treatments for 72 h (Figure 9). The MK-4 and MKH-SUC treatments also induced cleaved caspase-3 and PARP at 48 h, and pro-caspase 3 decreased at 72 h (Figure 9). The expression of cleaved PARP was lower in HL-60R cells than in HL60 cells (Figure 9). The MKH-DMG treatment did not affect the levels of these proteins (Figure 9).

## 4. Discussion

MK-4 is used to treat osteoporosis at a dose of 45 mg/day in Japan, and in this dosage regimen, the maximum blood level of MK-4 is 1 µM [23]. In distribution studies using rats and dogs, the levels of MK-4 in the bone marrow were approximately 20 and 2–3 times greater than in plasma, respectively [24,25]. The level of MK-4 in the bone marrow was comparable with the IC_50_ values of MKH-DMG and MHK-SUC in HL60 or HL-60R cells (Table 1). In addition, treatment with MKH derivatives would be safer because they are eliminated through a metabolic pathway, the vitamin K cycle, similar to MK-4 administration following MKH delivery. Thus, it is strongly suggested that MKH-DMG and MKH-SUC could act as chemotherapeutic agents with a long-term safety profile. Furthermore, MK-4 is practically insoluble, or insoluble in water, according to the monograph of Japanese Pharmacopoeia 17th edition, whereas MKH derivatives are highly water-soluble. Thus, MKH derivatives are injectable intravascularly and intrathecally without surfactants.

First, we examined the growth-inhibitory effects of the MKH derivatives MKH-DMG and MKH-SUC on HL60 and HL-60R cells (Figure 2B,D and Table 1) and determined intracellular MKO and MK-4 levels. Both MKH-DMG and MKH-SUC were effective on not only HL60 but also HL-60R cells, and their effects were more potent than those of MK-4. Although the curves between cells treated with each drug were significantly different at a significance level of 0.05, the resistance index values of the MKH derivatives were close to 1 and considerably lower than those of ATRA and MK-4 (Table 1), indicating the potential to overcome ATRA resistance. In addition, MKH-DMG and MKH-SUC exhibited similar effects on the APL cell line NB-4 (Supplementary Figure S1). Therefore, MKH-DMG and MKH-SUC are potentially effective against ATRA-resistant APL, and it is necessary to evaluate their efficacy against ATRA-resistant APL cell lines. Moreover, it is important to clarify the non-specificity to HL60 by observing the effect of MKH derivatives on multiple hematological cancer cells. Under the MK-4 treatment, the saturation of intracellular MKO and MK-4 levels was observed in both cell lines (Figure 3A–D). Thus, the phenomenon may limit its inhibitory effects (Figure 2B). In contrast, because MKH-SUC efficiently delivered MKH to both cell lines without any saturation (Figure 3A–D and Figure 4A–D), MKH-SUC may exert strong growth- inhibitory effects against both cell lines (Figure 2D) compared with the MK-4 treatment (Figure 2B). Interestingly, although MKH delivery (intracellular MKO and MK-4 levels) after MKH-DMG treatment was lower than that after MK-4 treatment (Figure 4A–D and Table 2), MKH-DMG showed a strong growth inhibitory effect against both cell lines (Figure 2C). Intracellular ester forms containing monoesters (1-mono and 4-mono) and 1,4-bis-ester (Supplementary Figure S2) probably affect MKH-DMG-mediated inhibition because the rate of intracellular hydrolysis of MKH-DMG must be lower than that of MKH-SUC, similar to the findings of our previous studies using different cell types [18,22]. For intracellular drug extraction, the cells were centrifuged in PBS and washed twice. Consequently, well-fragmented cells may be removed; however, damaged/dying cells may remain in the cell sample used for drug extraction. As the inhibitory effects of MKH derivatives were not observed until after 48 h, the irregular behavior of intracellular MK-4 and MKO from 48 h to 72 h after treatment with MKH-SUC and MKH-DMG may have been affected by cell toxicity (Figure 4A–D).

It has been demonstrated that ATRA induces apoptosis and cell cycle arrest along with cell differentiation [7,21,26,27]. In the present study, G_1_ arrest was observed in HL60 cells treated with ATRA (Figure 5A); however, this arrest may have been masked by ATRA resistance in the HL-60R cells (Figure 5B). It has also been reported that MK-4 induces apoptosis, cell cycle arrest with differentiation, and autophagic cell death against APL-derived cell lines [9,11,12,13,14,15,16]. Nevertheless, in this study, both MK-4 and MKH derivatives did not affect cell cycle arrest (Figure 5C–H). Miyazawa et al. demonstrated, in HL60 cells overexpressing Bcl-2, an apoptosis-inhibitory protein, that the primary growth-inhibitory effect of MK-4 is the induction of apoptosis rather than differentiation [13]. Similar to previous reports, the MK-4 and MKH-SUC treatments led to an increase in the number of cells in the sub-G_1_ phase (Figure 5C,D,G,H), along with increasing annexin V-positive cells (Figure 6C,D). Based on these results, we conclude that MK-4 and MKH-SUC exert a growth-inhibitory effect by inducing apoptosis and masking these effects on the cell cycle. Furthermore, Karasawa et al. demonstrated that MK-4-induced apoptosis is dependent on covalently bound MKO and BAK [12]. In the present study, the MK-4 and MKH-SUC treatments induced BAK protein modification and activated caspase-3 (Figure 9). These results indicate that apoptosis induced by MK-4 and MKH-SUC is mitochondrially mediated through BAK modification. As we did not isolate MKO from the shifted BAK protein band, it is unclear whether MKO is covalently bound to BAK. Further studies are therefore needed to clarify this issue. Moreover, a decrease in mitochondrial membrane potential caused by MK-4 and MKH-SUC was observed (Figure 7). Mitochondrial cytochrome c was released from the mitochondria to the cytosol by MKH-SUC treatment (Figure 8). However, the cytosolic cytochrome c level was not increased by MK-4 (Figure 8). It is speculated that cytosolic cytochrome c may have been consumed because it was bound to apoptosis protease-activating factor 1 (APAF-1) to promote apoptotic signaling [28,29]. Although MKH-DMG showed a strong growth inhibitory effect similar to MKH-SUC, it induced a small percentage of sub-G1 stage cells (Figure 5E,F), few apoptotic cells (Figure 6C,D), and no mitochondria-mediated apoptosis through BAK activation (Figure 7, Figure 8 and Figure 9), suggesting the involvement of another mechanism independent of apoptosis. As mentioned earlier, because MK-4 induces autophagic cell death [15], the inhibitory effect of MKH-DMG may involve autophagy. Further clarification of the mechanisms underlying the inhibitory effects of MKH derivatives, including the mechanism of how MKH derivatives avoid ATRA resistance, is needed.

## 5. Conclusions

The MKH derivatives (MKH-SUC and MKH-DMG) showed potential to be developed as APL therapeutic drugs that overcome ATRA resistance. Although MKH-SUC enhanced MKH delivery to cells compared with MK-4, MKH-DMG exerted an inhibitory effect owing to the intracellular delivery of ester forms (bis-ester and/or monoesters) rather than efficient MKH delivery, suggesting another unique mechanism independent of apoptosis. To develop a novel and safe chemotherapeutic agent effective against ATRA-resistant cell lines using MKH derivatives, further assessment of the mechanisms underlying the growth-inhibitory effect of MKH derivatives involved in ATRA resistance and in vivo pharmacological studies are required.

## Figures and Tables

**Table 1:** IC_50_ values of ATRA, MK-4, and MKH-ester derivatives in HL60 or HL-60R cells after 72 h.

Compound	IC_50_ (µM) (95% CI ^a^)		Resistance Index ^b^
	HL60 Cells	HL-60R Cells	
ATRA	1.15 (0.416–2.52)	7.83 (4.72–13.0)	6.79
MK-4	8.33 ^c^ (5.25–13.2)	32.5 ^c^ (20.2–52.3)	3.94
MKH-DMG	4.30 (2.89–6.40)	5.97 (3.74–9.53)	1.39
MKH-SUC	3.58 (2.66–4.83)	5.14 (2.81–9.40)	1.44

^a^ 95% Confidence interval. ^b^ Resistance index = IC_50_ for HL-60R/IC_50_ for HL60. ^c^ IC_50_ values for MK-4 were considered just for reference.

**Table 2:** Area under the curve over 72 h (AUC_0–72h_) of intracellular concentrations of MK-4 and MKO in HL60 and HL-60R cells treated with 5 μM MK-4, MKH-DMG, or MKH-SUC.

Compound	AUC_0–72h_ ^a^ (nmol·h·mg Protein^−1^) in HL60 Cells		AUC_0–72h_ ^a^ (nmol·h·mg Protein^−1^) in HL-60R Cells	
	MK-4	MKO	MK-4	MKO
MK-4	26.4 ± 0.80	6.33 ± 0.32	19.9 ± 0.40	5.52 ± 0.03
MKH-DMG	12.3 ± 1.29	3.11 ± 0.36	7.90 ± 0.66	2.29 ± 0.15
MKH-SUC	38.6 ± 0.50	9.10 ± 0.22	30.6 ± 1.24	8.70 ± 0.46

^a^ Values are shown as mean ± SD (n = 3).

## Data Availability

The data that support the findings of this study are available from the corresponding author upon reasonable request.

## Supplementary Materials

The following are available online at https://www.mdpi.com/article/10.3390/pharmaceutics13050758/s1, Figure S1: Inhibitory effects of MKH derivatives on the viability of NB-4 cells; Figure S2: Intracellular concentration of MKH-esters in HL60 and HL-60R treated with MKH-DMG; Table S1: Area under the curve over 72 h (AUC_0–72h_) of intracellular concentrations of MKH-esters in HL60 and HL-60R cells treated with 5 μM MKH-DMG.
