# Supplementary material for: Growth Inhibitory Effects of Ester Derivatives of Menahydroquinone-4, the Reduced Form of Vitamin K2(20), on All-Trans Retinoic Acid-Resistant HL60 Cell Line
Source: Pharmaceutics. 2021 May 20;13(5):758. doi: 10.3390/pharmaceutics13050758 (PMC8161027; doi:10.3390/pharmaceutics13050758)
Supplement: Supplementary file 1 [file pharmaceutics-13-00758-s001.zip › pharmaceutics-1093390-supplementary.pdf]

pharmaceutics

Supplementary Materials: Growth Inhibitory Effects of Ester Derivatives of Menahydroquinone-4, the Reduced Form of Vitamin $K_{2(20)}$, on All-Trans Retinoic Acid-Resistant HL60 Cell Line

Hirofumi Yamakawa, Shuichi Setoguchi, Shotaro Goto, Daisuke Watase, Kazuki Terada, Nami Nagata-Akaho, Erina Toki, Mitsuhisa Koga, Kazuhisa Matsunaga, Yoshiharu Karube and Jiro Takata

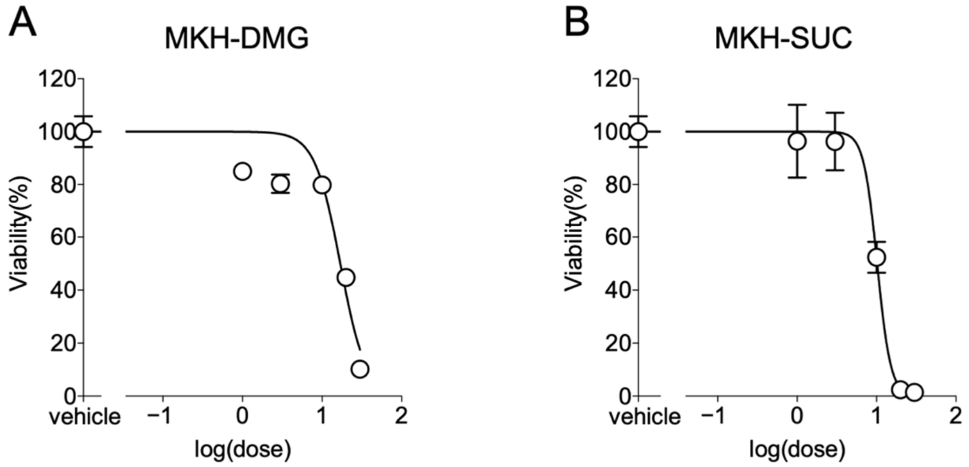

Figure S1. Inhibitory effects of MKH derivatives on the viability of NB-4 cells. The human promyelocytic cell lines NB-4 were provided by Cell Line Service (Eppelheim, German). The cells were treated with 1–30 µM MKH-DMG (**A**) or MKH-SUC (**B**) for 72 h. This experiment was performed as described in Materials and Methods 2.3. Error bars indicate mean ±SD (*n* = 3).

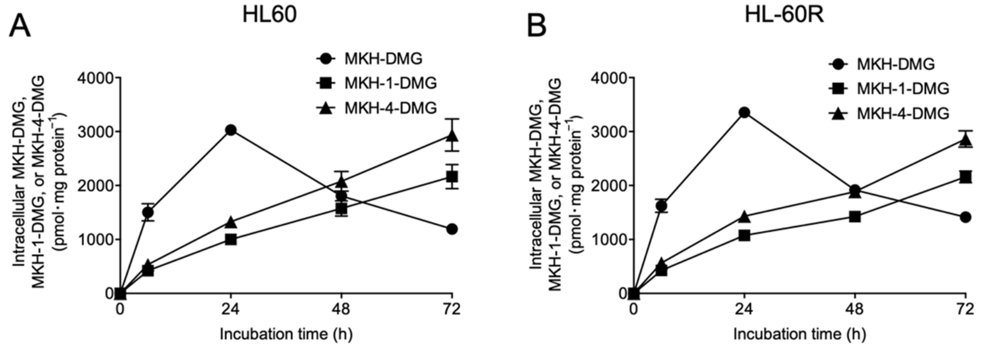

Figure S2. Intracellular concentration of MKH-esters (1,4-bis-ester, 1-monoester, and 4-monoester) in HL60 (**A**) and HL-60R (**B**) cells treated with 5 µM MKH-DMG up to 72 h. Error bars indicate mean ± SD (*n* = 3).

Table S1. Area under the curve over 72 h ($AUC_{0–72\ h}$) of intracellular concentrations of MKH-esters (1,4-bis-ester, 1-monoester, and 4-monoester) in HL60 and HL-60R treated with 5 μM MKH-DMG.

**Intracellular Concentrations of MKH-Esters**	**$AUC_{0-72\ h}$[a] (nmol·h·mg protein^{-1})**	
	**HL60**	**HL-60R**
MKH-DMG	139 ± 5.54	153 ± 3.08
MKH-1-DMG	89.9 ± 6.15	87.8 ± 4.21
MKH-4-DMG	119 ± 7.95	116 ± 5.65

[a] values are shown as Mean ± SD (*n* = 3).
